# Interference Between DNA Replication and Transcription as a Cause of Genomic Instability

**DOI:** 10.2174/138920212799034767

**Published:** 2012-03

**Authors:** Yea-Lih Lin, Philippe Pasero

**Affiliations:** Institute of Human Genetics, CNRS-UPR1142, Montpellier, France

**Keywords:** DNA polymerases, genomic instability, replication, R-loops, RNase H, RNA polymerases, transcription.

## Abstract

Replication and transcription are key aspects of DNA metabolism that take place on the same template and potentially interfere with each other. Conflicts between these two activities include head-on or co-directional collisions between DNA and RNA polymerases, which can lead to the formation of DNA breaks and chromosome rearrangements. To avoid these deleterious consequences and prevent genomic instability, cells have evolved multiple mechanisms preventing replication forks from colliding with the transcription machinery. Yet, recent reports indicate that interference between replication and transcription is not limited to physical interactions between polymerases and that other cotranscriptional processes can interfere with DNA replication. These include DNA-RNA hybrids that assemble behind elongating RNA polymerases, impede fork progression and promote homologous recombination. Here, we discuss recent evidence indicating that R-loops represent a major source of genomic instability in all organisms, from bacteria to human, and are potentially implicated in cancer development.

## INTRODUCTION

DNA replication and transcription are fundamental genetic processes that need to be tightly regulated and coordinated to preserve the integrity of the genome and to promote its faithful transmission to daughter cells. These processes involve large protein complexes progressing at high speed and for long distances along the chromosomes. Under normal growth conditions, the genome is most vulnerable during the S phase of the cell cycle. Indeed, the replication machinery must overcome obstacles such as abasic sites, secondary DNA structures or tightly-bound protein complexes, which can cause replication forks stalling [1, 2]. Arrested forks are fragile structures that are prone to recombination and chromosomal rearrangements [3, 4]. A large body of evidence from prokaryotes and eukaryotes indicates that encounters between DNA and RNA polymerases can also induce replication fork arrest [5, 6] and genomic instability [7-12]. Mechanisms that limit interference between DNA replication and transcription are therefore important for the maintenance and the faithful duplication of eukaryotic and prokaryotic genomes. In this review, we focus on the nature of this interference and its consequence upon genome integrity. 

## INTERFERENCE BETWEEN REPLICATION AND TRANSCRIPTION IN BACTERIA

**Fig. (1):**
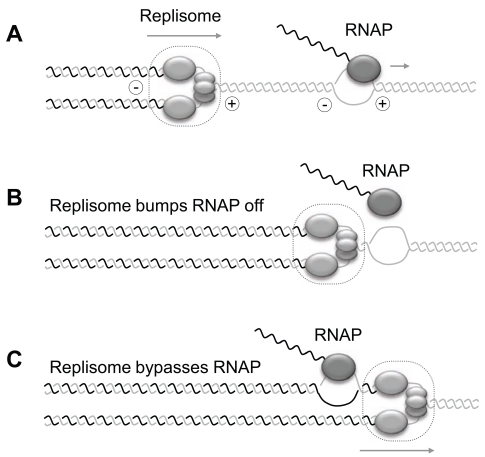
Model for co-directional collision between transcription and replication machineries. (**A**) Co-directional progression of replication and transcription machineries. (+) and (-) signs indicate the formation of positive and negative DNA supercoiling ahead and behind polymerase complexes, respectively. This event is extremely frequent in prokaryotes due to the fact that the replisome is much faster than RNA polymerases (RNAP), but does not significantly affect fork progression. Two models account for the bypass of transcription complexes by replication forks. (**B**) The replisome displaces the RNAP from the DNA template and uses the RNA transcript as a primer to continue leading-strand synthesis [24]. (**C**) The replisome bypasses RNAP without arresting transcription [29].

**Fig. (2):**
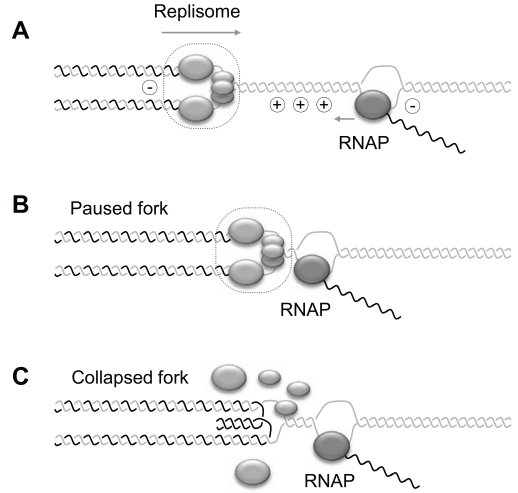
Model for head-on collision between transcription and replication machineries. (**A**) Convergence of the two polymerase complexes along the template leads to the accumulation of positive DNA supercoiling (+). This topological constraint contributes to the interference between converging replication and transcription machineries [68]. (**B**) Head-on collision between DNA and RNA polymerases and/or accumulation of DNA supercoiling induces a pausing of the replication fork [4]. (**C**) Paused forks are fragile structures that can be converted into collapsed forks and induce HR upon disassembly of the replisome and/or fork reversion [3, 68].

Many aspects of the organization of bacterial genomes are conserved and are important for cell survival. For instance, chromosomal rearrangements such as large inversions can lead to impaired growth or cell death [13-16]. Almost 25 years ago, it has been proposed that one benefit of proper genomic organization may be the reduction of potential conflicts between replication and transcription by limiting frontal collisions between replisomes and RNA polymerases (RNAPs) [17-20]. Since the same DNA template is used for replication and transcription, collisions occur when polymerases move towards each other (head-on collision) or move in the same direction (co-directional collision). In bacteria, co-directional collision is a frequent event as replication forks move 12-30 times faster than transcription complexes [21-23]. Replisomes are therefore expected to overtake RNAPs moving in the same direction. Yet, co-directional collisions are less harmful than head-on collisions and can be resolved in two ways (Fig. **1**). The replication complex can bump the RNAP off the DNA template, resulting in the premature abrogation of transcription. Alternatively, the replication fork can pass RNAP ternary complex without arresting transcription [24-26]. In both cases, co-directional collisions have little or no effect on replication fork progression. In contrast, the replisome pauses when it encounters a RNAP moving in the opposite direction (Fig. **2**). This view is supported by electron microscopy studies showing that replication forks progressing through a highly-transcribed rRNA operon are slower when they run against the direction of transcription [27]. Extensive analyses of frontal encounters between the DNA replication apparatus of bacteriophage T4 and RNAP ternary complex from *E. coli *also revealed that the replisome pauses for a few seconds after encountering the RNAP. This pause extends for up to several minutes in the absence of accessory helicase [8]. Other studies indicate that paused replisomes remain stable and eventually resume elongation after displacing RNAP from the DNA template [28, 29].

In *Bacillus subtilis*, most of the genes (75%) are co-oriented with replication [30]. Using a genome-wide approach to monitor the effects of altering the co-orientation bias of transcription and replication on fork progression, Wang and colleagues have found that this co-orientation reduces adverse effects of transcription on DNA replication. They showed that in wild-type cells (75% co-orientation), replication proceeds without detectable interference with transcription. In contrast, replication elongation is impeded in the regions with reversed bias. The reduction in replication elongation is detected throughout the genome and is not limited to highly expressed genes, such as the rRNA operons [31]. Yet, the severity of replication impediment due to head-on collisions with transcription seems to correlate with the level of gene expression. Moreover, artificial reversion of ribosomal genes orientation induced a SOS response, genomic instability and cell death [32]. These data support the view that bacterial genomes have evolved to minimize head-on collision between replisome and transcription machinery. This co-directional organization of DNA replication and highly-expressed genes is conserved in all known bacteria [33]. 

## INTERFERENCE BETWEEN REPLICATION AND TRANSCRIPTION IN EUKARYOTES

Interference between replication and transcription has been extensively studied in the budding yeast *Saccharomyces cerevisiae *[5, 34]. One of the best-characterized examples of conflicts between RNA and DNA polymerases is found at ribosomal RNA genes array (rDNA). This locus contains ~ 150 copies of 35S rRNA genes that are transcribed by RNA polymerase I throughout the cell cycle [35]. Each rDNA unit contains a replication origin, but only 20% of these origins are used every cell cycle [19, 36]. Active origins are located downstream of active 35S genes and form clusters of 3 to 5 adjacent units, separated by each other with large domains devoid of active origins [37, 38]. Replication of the rDNA array represents a real challenge for replisomes proceeding opposite to the direction of transcription. Using bidimensional agarose gel electro-phoresis, the Fangman and Huberman laboratories have shown that a polar replication fork barrier (RFB) located downstream of 35S genes arrests forks progressing opposite to the direction of RNA pol I transcription [19, 36]. Replication fork arrest at the rDNA depends on Fob1, a non-essential and poorly-characterized protein interacting with RFBs [39, 40]. Surprisingly, deletion of the *FOB1* gene does not increase head-on collisions between DNA and RNA polymerases in normal growth conditions. Indeed, collisions are only detected if the number of rDNA units is reduced to 20 copies in order to increase the rate of transcription at individual genes [41]. These data argue against the view that the primary role of RFBs is to prevent interference between replication and transcription at the 3’ end of 35S genes.

A large body of evidence indicates that fork arrest at RFBs induces DSBs, promotes homologous recombination at the rDNA array and determines the replicative lifespan of *S. cerevisiae *[42-46]. RFB-dependent recombination is stimulated by Fob1 and repressed by the histone deacetylase Sir2, another key determinant of replicative lifespan in budding yeast [42, 47-50]. It is generally believed that unequal sister chromatid recombination in the rDNA is essential to maintain the length and the homogeneity of the array [1, 51]. The mechanisms through which Fob1 and Sir2 modulate rDNA recombination are not fully understood, but involve alterations of sister-chromatid cohesion at arrested forks [52, 53]. Altogether, these data suggest that the rDNA RFB evolved from a simple structure preventing head-on collisions between polymerases to a more complex system ensuring the maintenance and the homeostasis of the rDNA array. Interestingly, replication fork arrest at RFBs does not activate a checkpoint response, presumably because the amount of ssDNA exposed at RFB-arrested forks is limited to a few nucleotides [54]. Replication fork barriers are also present at human and mouse rRNA genes [55, 56]. Unlike in *S. cerevisiae*, the human rDNA array contains rRNA gene palindromes that are particularly difficult to replicate and induce genomic instability in cancer cell lines [57].

Besides rRNA genes, polar replication pause sites have also been reported at tRNA genes in budding yeast [11, 58]. As for rDNA RFBs, tRNA genes only arrest replication forks when they oppose the direction of transcription. Fork pausing is not detected in conditional mutants defective for transcription initiation, suggesting that fork arrest and transcriptional activity are mechanistically linked [11]. Interestingly, a recent report shows that the DNA replication checkpoint down-regulates transcription at tRNA genes through the dephosphorylation of the transcriptional repressor Maf1 [59]. It is tempting to speculate that replication/transcription interference at tRNA genes activates a checkpoint that relieves fork arrest by inhibiting transcription at tRNA genes. Further experiments are required to address this possibility.

Experimental evidence indicates that RNA polymerase II can also induce a replication fork pause upon ‘head-on’ collision with oncoming replication forks, whereas co-directional transcription has little effect on fork progression [60]. Using a microarray-based approach, Azvolinsky *et al.* have recently shown that highly-transcribed genes are enriched in DNA polymerase in exponentially-growing cells [61], indicating that replication forks slow down when they frequently encounter RNAPs. DNA pol enrichment is independent of gene orientation in this assay, which argues against the view that fork pausing is mostly caused by frontal collisions. However, it is worth mentioning that unlike bacteria, eukaryotes initiate replication from multiple and relatively inefficient origins [62]. Most eukaryotic genes are therefore replicated from both directions, which could explain why fork pausing is apparently independent of gene orientation at the genome-wide level.

Both transcription and replication have profound effects on DNA topology. During transcription, RNAP is prevented from rotating along the helical axis of the DNA template by processing factors present on nascent RNA. This leads to the accumulation of positive and negative DNA supercoiling ahead and behind the enzyme, respectively [63]. Since positive DNA supercoiling also accumulates in front of the replication fork [64], this torsional stress increases when RNA and DNA polymerases converge (Fig. **2A**), which can have direct consequences on both processes [65-67]. In the case of DNA replication, positive DNA supercoiling can also lead to the arrest and/or the reversal of replication forks [4, 12, 68, 69]. This represents a potential source of genomic instability, as discussed in the following sections.

It is not clear whether the collinear organization of replication and transcription is conserved in eukaryotic cells, as it is the case in bacteria. In budding yeast, a study carried out to determine the directions of transcription and replication in 137 ribosomal protein genes - which account for ~ 50% of the transcription by RNA polymerase II - revealed no significant correlation [70]. In human, computational predictions of origins of replication based on the nucleotide compositional skew of the genome suggested that a large fraction of highly-expressed genes are co-oriented with replication fork progression [71]. However, this view has recently been challenged by the analysis of a large dataset of experimentally-determined replication origins in human cells, which failed to detect a significant gene orientation bias in the proximity of replication origins [72-74]. The recent release of the first complete datasets of replication origins in other multicellular organisms [75] will certainly help clarify this issue.

Another specificity of eukaryotic cells that contributes to limit replication/transcription interference is the spatial and temporal compartmentalization of the nucleus. Indeed, DNA replication, transcription, RNA maturation and export occur at distinct sites in the nucleus, which segregate into higher-order domains and display a network-like appearance [76-79]. These sites remain spatially distinct throughout the length of the S phase, only 3% of the replication foci overlapping with transcription sites in early S phase [79]. These data suggest that different replication and transcription domains are progressively activated and inactivated as the cell traverses S phase, following temporal programs of replication and transcription [80]. An attractive possibility is that higher eukaryotes have evolved this functional organization to restrain interference between replication and transcription and to maintain the integrity of their genome. Yet, reports presented below indicate that conflicts between replication and transcription occur anyhow in eukaryotic cells and promote genomic instability.

## FUNCTIONAL LINKS BETWEEN TRANSCRIPTION, REPLICATION AND RECOMBINATION

Homologous recombination (HR) is critical for the maintenance of genome integrity. Yet, uncontrolled HR is also largely responsible for the chromosomalre arrangements detected in cancer cells [81]. The mechanisms that promote HR in normal growth conditions are poorly understood. A large body of evidence from both prokaryotes and eukaryotes indicates that transcription plays a central role in the induction of spontaneous genomic instability [7, 34, 82, 83]. This phenomenon, termed transcription-associated recombination (TAR), is involved in developmentally-regulated processes such as class switch recombination of immunoglobulin (Ig) genes [5, 84, 85]. Since stalled replication forks trigger HR [1, 3, 4], a likely possibility is that transcription promotes genomic instability by blocking replication forks [7, 34]. This is indeed the case in *S. cerevisiae* cells, head-on collision between the replisome and RNAP increasing both fork pausing and HR between direct repeats [5]. Importantly, HR does not increase in the absence of replication or when replisomes and RNAPs go in the same direction [60]. In line with these observations, HR is also associated with gene expression and DNA replication in mammalian cells [86]. Interestingly, TAR is associated with signatures of one-ended double-strand break recombination and not with classical two-ended DSB repair [87]. These data support the view that recombination is initiated at stalled forks in these cells and not at chromosome breaks.

Functional links between TAR and replication fork stalling have been consolidated by the observation that inhibition of messenger ribonucleo protein particles (mRNP) biogenesis in yeast *hpr1 *mutants impairs transcription, replication fork progression and drastically increases TAR [5, 88]. Hpr1 belongs to THO/TREX, a conserved complex acting at the interface between transcription and mRNP metabolism [10, 89, 90]. In a recent study, the Aguilera lab has shown that Hpr1 associates with active genes throughout the yeast genome, with a gradual enrichment at the 3’-end of transcription units [91]. This association is particularly important for the transcription of long, highly-expressed and GC-rich genes. In *hpr1* mutants, these genes show an increased recruitment of Rrm3, a specialized helicase implicated in the displacement of obstacles ahead of replication forks [58, 61]. Interestingly, overexpression of RNaseH1, an enzyme that degrades specifically RNA-DNA hybrids, reduces the recruitment of Rrm3 at these highly-expressed genes [91]. These data suggest that the THO/TREX complex prevents the formation of DNA-RNA hybrids during transcription by promoting the assembly of mRNPs on nascent RNAs. In *hpr1* mutants, defective mRNP assembly would interfere with replication fork progression and would induce the recruitment of Rrm3 at paused forks. Other evidence supporting the view that RNA-DNA hybrids interfere with DNA replication is presented below.

## R-LOOPS AND THEIR EFFECT ON REPLICATION FORK PROGRESSION

**Fig. (3):**
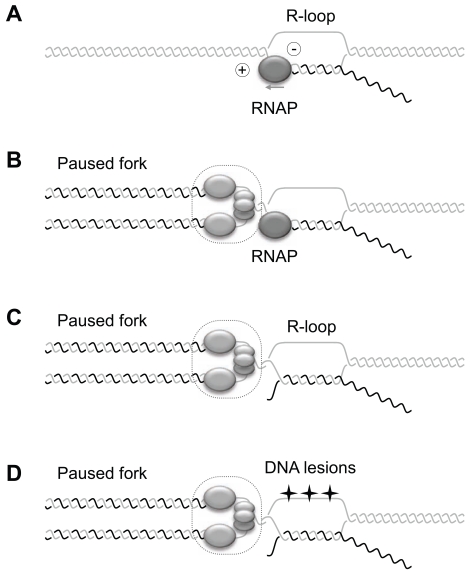
Model for interference between cotranscriptional R-loops and replication fork progression. (**A**) Cotranscriptional R-loops form during transcription when the nascent RNA anneals to the template DNA strand, leaving the non-template strand unpaired. R-loop formation is favored by the accumulation of negative supercoiling behind the RNA polymerase (-). Different mechanisms have been proposed to explain how RNA-DNA hybrids perturb fork progression [5, 7]. (**B**) R-loops may prevent the displacement of the RNAP upon passage of the forks. (**C**) The RNA/DNA hybrid may directly prevent DNA synthesis on the leading and/or the lagging strand of the fork. (**D**) R-loops may interfere indirectly with DNA replication by favoring the accumulation of DNA lesions on the exposed ssDNA strand (stars).

Cotranscriptional R-loops form during transcription when the nascent RNA anneals to the template DNA strand, leaving the non-template strand unpaired (Fig. **3A**). Studies in bacteria have shown that formation of RNA-DNA hybrids occurs preferentially at GC-rich regions and is favored by the accumulation of negatively supercoiled DNA behind the advancing RNA polymerase II [65]. Besides their involvement in class switch recombination of mammalian immunoglobulin genes [92, 93], a growing body of evidence indicates that R-loops affect the integrity of eukaryotic genomes by blocking replication forks. In yeast THO/TREX mutants, accumulation of R-loops behind elongating RNA polymerase II is the major cause of impaired transcription and increased recombination [94]. Interestingly, replication fork stalling is suppressed by the overexpression of RNase H, an enzyme that degrades R-loops [5]. Moreover, one particular mutant of THO/TREX complex increasing transcription defects but not R-loop formation, does not induce fork arrest and TAR [95]. Collectively, these data indicate that R-loops increase TAR in budding yeast by impeding replication fork progression. 

A growing body of evidence indicates that similar TAR mechanisms operate in higher eukaryotes. In chicken DT40 cells, depletion of the splicing factor ASF/SF2 induces the formation of RNA-DNA hybrids and increases genomic instability [96]. As in yeast THO/TREX mutants, genomic instability in ASF/SF2-deficient cells is suppressed by the over-expression of RNase H [96]. Other studies in mammalian cells indicate that TAR depends on DNA replication as it is only detected in S-phase cells [7]. This functional link is further strengthened by the recent observation that depletion of DNA Topoisomerase I (Top1) in mammalian cells induces replication fork stalling and spontaneous DNA damage at highly-expressed genes. Fork arrest in Top1-deficient cells is largely due to the accumulation of R-loops during transcription as replication impediments are largely suppressed by RNase H overexpression. Top1 displays a kinase activity that is implicated in the regulation of splicing factors of the SR family such as ASF/SF2 [97]. It is therefore likely that Top1 prevents interference between replication and transcription not only by relaxing topological stress at converging RNA and DNA polymerases, but also by promoting the ASF/SF2-dependent inhibition of R-loops formation [12]. Interestingly, several other mRNA-processing enzymes have recently been identified in a genome-wide screen for factors required to prevent spontaneous chromosome breaks [98]. Increased genomic instability and chromosome breaks were also reported in OMCG1-deficient mouse fibroblasts [99]. In both studies, chromosome breaks were at least partially suppressed by the overexpression of RNase H, supporting the view that multiple factors cooperate to prevent the formation of R-loops.

It is now well established that replication fork arrest activates the DNA replication response, a checkpoint pathway that is essential for the maintenance of paused forks [1, 4]. Since co-transcriptional R-loops impair replication fork progression, an important question is whether these structures also activate the DNA replication checkpoint. This issue has recently been addressed in yeast and murine cells [99, 100]. In yeast *hpr1* mutants, the Mec1-Rad53 pathway is constitutively activated, supporting the view that R-loops-mediated fork stalling activates the S-phase checkpoint. This pathway is also important for *hpr1 *mutants survival under replication stress [100]. In mouse embryonic fibroblasts, OMCG1 is a target of the ATR/ATM checkpoint kinases [99] and is essential for S phase progression [101]. OMCG1 depletion induces genomic instability and checkpoint activation. These genomic alterations can be significantly relieved by the overexpression of RNaseH indeficient fibroblasts, suggesting that R-loops formation contributes to replication stress and checkpoint activation in these cells [99]. 

Despite the growing literature on R-loops and genomic instability, very little is known on the mechanism(s) by which these RNA/DNA hybrids form and interfere with replication forks. Indeed, the mapping of these structures is technically challenging and has been limited so far to a small number of loci in eukaryotic cells [96]. Moreover, biochemical characteristics such as the length and the stability of R-loops in the context of chromatin are unclear [102]. In principle, RNA-DNA hybrids could impede fork progression in three non-mutually exclusive ways (Fig. **3**). R-loops could interfere with replication by (*i*) preventing DNA synthesis on the leading or the lagging strand, (*ii*) preventing the displacement of the RNAP upon passage of the fork or (*iii*) promoting the accumulation of DNA lesions on the non-template ssDNA, which would in turn affect DNA synthesis [5]. In any case, RNA-DNA hybrids are expected to be particularly stable to resist the confronted replication machinery and its associated helicases. These structures could also interfere with DNA replication long after transcription has ceased. Two recent studies showing that the Sen1/Sentaxin helicase is involved in the resolution of R-loops in yeast and human cells will certainly be valuable to understand the metabolism of cotranscriptional RNA-DNA hybrids [103, 104]. Other candidates for the regulation of R-loops include the RecQ helicase RecQ5, which interacts directly with the C-terminal domain of RNA polymerase II and is important for the maintenance of genome integrity [105-107].

## CONCLUSION AND PERSPECTIVES

Transcription and DNA replication need to be tightly regulated to ensure the preservation of genome integrity and to promote faithful genome transmission to daughter cells. Increasing evidence from prokaryotes and eukaryotes indicates that cotranscriptional R-loops interfere with replication fork progression and represent a major source of genomic instability. This finding has major implication for current models of oncogene-induced tumorigenesis. Indeed, it is now well established that cells are exposed to chronic replication stress during the early stages of the cancer process [108]. However, the origin of this replication stress is currently unknown. Since deregulated oncogenes also affect patterns of gene expression, it is tempting to speculate that cotranscriptional R-loops contribute to the replication stress observed in oncogene-activated cells [109]. Further effort is needed to address this possibility, for instance through the development of novel genome-wide assay to detect RNA-DNA hybrids in the genome of pretumoral cells.

## References

[1] Tourriere H, Pasero P (2007). Maintenance of fork integrity at damaged DNA and natural pause sites. DNA Repair (Amst).

[2] Gangloff S, Soustelle C, Fabre F (2000). Homologous recombination is responsible for cell death in the absence of the Sgs1 and Srs2 helicases. Nat. Genet.

[3] Lambert S, Froget B, Carr A M (2007). Arrested replication fork processing: Interplay between checkpoints and recombination. DNA Repair.

[4] Branzei D, Foiani M (2010). Maintaining genome stability at the replication fork. Nat. Rev. Mol. Cell. Biol.

[5] Aguilera A, Gomez-Gonzalez B (2008). Genome instability: a mechanistic view of its causes and consequences. Nat. Rev. Genet.

[6] Mirkin EV, Mirkin S M (2005). Mechanisms of transcription-replication collisions in bacteria. Mol. Cell. Biol.

[7] Gottipati P, Helleday T (2009). Transcription-associated recombination in eukaryotes: link between transcription, replication and recombination. Mutagenesis.

[8] Liu B, Alberts B M (1995). Head-on collision between a DNA replication apparatus and RNA polymerase transcription complex. Science.

[9] Vilette D, Uzest M, Ehrlich S D, Michel B (1992). DNA transcription and repressor binding affect deletion formation in Escherichia coli plasmids. EMBO J.

[10] Chavez S, Garcia-Rubio M, Prado F, Aguilera A (2001). Hpr1 is preferentially required for transcription of either long or G+C-rich DNA sequences in Saccharomyces cerevisiae. Mol. Cell. Biol.

[11] Deshpande A M, Newlon C S (1996). DNA replication fork pause sites dependent on transcription. Science.

[12] Tuduri S, Crabbe L, Conti C, Tourriere H, Holtgreve-Grez H, Jauch A, Pantesco V, De Vos J, Thomas A, Theillet C, Pommier Y, Tazi J, Coquelle A, Pasero P (2009). Topoisomerase I suppresses genomic instability by preventing interference between replication and transcription. Nat. Cell. Biol.

[13] Hill C W, Gray J A (1988). Effects of chromosomal inversion on cell fitness in Escherichia coli K-12. Genetics.

[14] Rebollo J E, Francois V, Louarn J M (1988). Detection and possible role of two large nondivisible zones on the Escherichia coli chromosome. Proc. Natl. Acad. Sci. U S A.

[15] Campo N, Dias M J, Daveran-Mingot M L, Ritzenthaler P, Le Bourgeois P (2004). Chromosomal constraints in Gram-positive bacteria revealed by artificial inversions. Mol. Microbiol.

[16] Wu L J, Errington J (2002). A large dispersed chromosomal region required for chromosome segregation in sporulating cells of Bacillus subtilis. EMBO J.

[17] Nomura M, Morgan E A, Jaskunas S R (1977). Genetics of bacterial ribosomes. Annu. Rev. Genet.

[18] Rocha E R, Smith C J (2004). Transcriptional regulation of the Bacteroides fragilis ferritin gene (ftnA) by redox stress. Microbiology.

[19] Brewer B J, Fangman W L (1988). A replication fork barrier at the 3' end of yeast ribosomal RNA genes. Cell.

[20] Price M N, Alm E J, Arkin A P (2005). Interruptions in gene expression drive highly expressed operons to the leading strand of DNA replication. Nucleic Acids Res.

[21] Hwang D S, Kornberg A (1992). Opening of the replication origin of Escherichia coli by DnaA protein with protein HU or IHF. J. Biol. Chem.

[22] Mirkin E V, Mirkin S M (2007). Replication fork stalling at natural impediments. Microbiol. Mol. Biol. Rev.

[23] Breier A M, Weier H U, Cozzarelli N R (2005). Independence of replisomes in Escherichia coli chromosomal replication. Proc. Natl. Acad. Sci. U S A.

[24] Pomerantz R T, O'Donnell M (2008). The replisome uses mRNA as a primer after colliding with RNA polymerase. Nature.

[25] Liu B, Wong M L, Tinker R L, Geiduschek E P, Alberts B M (1993). The DNA replication fork can pass RNA polymerase without displacing the nascent transcript. Nature.

[26] Liu B, Wong M L, Alberts B (1994). A transcribing RNA polymerase molecule survives DNA replication without aborting its growing RNA chain. Proc. Natl. Acad. Sci. U S A.

[27] French S (1992). Consequences of replication fork movement through transcription units *in vivo*. Science.

[28] Pomerantz R T, O'Donnell M (2010). Direct restart of a replication fork stalled by a head-on RNA polymerase. Science.

[29] Pomerantz R, O’Donnell M (2010). What happens when replication and transcription complexes collide?. Cell Cycle.

[30] Kunst F, Ogasawara N, Moszer I, Albertini A M, Alloni G, Azevedo V, Bertero M G, Bessieres P, Bolotin A, Borchert S, Borriss R, Boursier L, Brans A, Braun M, Brignell S. C, Bron S, Brouillet S, Bruschi C. V, Caldwell B, Capuano V, Carter N. M, Choi S.-K, Codani J.-J, Connerton I. F, Cummings N. J, Daniel R. A, Denizot F, Devine K. M, Düsterhöft A, Ehrlich S. D, Emmerson P. T, Entian K. D, Errington J, Fabret C, Ferrari E, Foulger D, Fritz C, Fujita M, Fujita Y, Fuma S, Galizzi A, Galleron N, Ghim S.-Y, Glaser P, Goffeau A, Golightly E. J, Grandi G, Guiseppi G, Guy B. J, Haga K, Haiech J, Harwood C R, Hénaut A, Hilbert H, Holsappel S, Hosono S, Hullo M-F, Itaya M, Jones L, Joris B, Karamata D, Kasahara Y, Klaerr-Blanchard M, Klein C, Kobayashi Y, Koetter P, Koningstein G, Krogh S, Kumano M, Kurita K, Lapidus A, Lardinois S, Lauber J, Lazarevic V, Lee S-M, Levine A, Liu H, Masuda S, Mauël C, Médigue C, Medina N, Mellado R P, Mizuno M, Moestl D, Nakai S, Noback M, Noone D, O'Reilly M, Ogawa K, Ogiwara A, Oudega B, Park S-H, Parro V, Pohl T M, Portetelle D, Porwollik S, Prescott A M, Presecan E, Pujic P, Purnelle B, Rapoport G, Rey M, Reynolds S, Rieger M, Rivolta C, Rocha E, Roche B, Rose M, Sadaie Y, Sato T, Scanlan E, Schleich S, Schroeter R, Scoffone F, Sekiguchi J, Sekowska A, Seror S. J, Serror P, Shin B.-S, Soldo B, Sorokin A, Tacconi E, Takagi T, Takahashi H, Takemaru K, Takeuchi M, Tamakoshi A, Tanaka T, Terpstra P, Tognoni A, Tosato V. Uchiyama S, Vandenbol M, Vannier F, Vassarotti A, Viari A, Wambutt R, Wedler E, Wedler H, Weitzenegger T, Winters P, Wipat A, Yamamoto H, Yamane K, Yasumoto K, Yata K, Yoshida K, Yoshikawa H-F, Zumstein E, Yoshikawa H, Danchin A (1997). The complete genome sequence of the gram-positive bacterium Bacillus subtilis. Nature.

[31] Wang J D, Berkmen M B, Grossman A D (2007). Genome-wide coorientation of replication and transcription reduces adverse effects on replication in Bacillus subtilis. Proc. Natl. Acad. Sci. USA.

[32] Srivatsan A, Tehranchi A, acAlpine D M, Wang J D (2010). Co-orientation of replication and transcription preserves genome integrity. PLoS Genet.

[33] Guy L, Roten C A (2004). Genometric analyses of the organization of circular chromosomes: a universal pressure determines the direction of ribosomal RNA genes transcription relative to chromosome replication. Gene.

[34] Aguilera A (2002). The connection between transcription and genomic instability. EMBO J.

[35] Saffer L D, Miller OL (1986). Electron microscopic study of Saccharomyces cerevisiae rDNA chromatin replication. Mol. Cell. Biol.

[36] Linskens M H, Huberman J A (1988). Organization of replication of ribosomal DNA in Saccharomyces cerevisiae. Mol. Cell. Biol.

[37] Muller M, Lucchini R, Sogo J M (2000). Replication of yeast rDNA initiates downstream of transcriptionally active genes. Mol. Cell.

[38] Pasero P, Bensimon A, Schwob E (2002). Single-molecule analysis reveals clustering and epigenetic regulation of replication origins at the yeast rDNA locus. Genes Dev.

[39] Kobayashi T, Horiuchi T (1996). A yeast gene product, Fob1 protein, required for both replication fork blocking and recombinational hotspot activities. Genes Cells.

[40] Mohanty B K, Bastia D (2004). Binding of the replication terminator protein Fob1p to the Ter sites of yeast causes polar fork arrest. J. Biol. Chem.

[41] Takeuchi Y, Horiuchi T, Kobayashi T (2003). Transcription-dependent recombination and the role of fork collision in yeast rDNA. Genes Dev.

[42] Defossez P A, Prusty R, Kaeberlein M, Lin S J, Ferrigno P, Silver P A, Keil R L, Guarente L (1999). Elimination of replication block protein Fob1 extends the life span of yeast mother cells. Mol. Cell.

[43] Kaeberlein M, McVey M, Guarente L (1999). The SIR2/3/4 complex and SIR2 alone promote longevity in Saccharomyces cerevisiae by two different mechanisms. Genes Dev.

[44] Kim S, Benguria A, Lai C Y, Jazwinski S M (1999). Modulation of life-span by histone deacetylase genes in Saccharomyces cerevisiae. Mol. Biol. Cell.

[45] Sinclair D A, Mills K, Guarente L (1997). Accelerated aging and nucleolar fragmentation in yeast sgs1 mutants. Science.

[46] Fritsch O, Burkhalter M D, Kais S, Sogo J M, Schär P (2010). DNA ligase 4 stabilizes the ribosomal DNA array upon fork collapse at the replication fork barrier. DNA Repair.

[47] Johzuka K, Horiuchi T (2002). Replication fork block protein, Fob1, acts as an rDNA region specific recombinator in S. cerevisiae. Genes Cells.

[48] Kobayashi T, Heck D J, Nomura M, Horiuchi T (1998). Expansion and contraction of ribosomal DNA repeats in Saccharomyces cerevisiae: requirement of replication fork blocking (Fob1) protein and the role of RNA polymerase I. Genes Dev.

[49] Gottlieb S, Esposito R E (1989). A new role for a yeast transcriptional silencer gene, SIR2, in regulation of recombination in ribosomal DNA. Cell.

[50] Mayan-Santos M D, Martinez-Robles M L, Hernandez P, Schvartzman J B, Krimer D. B (2008). A redundancy of processes that cause replication fork stalling enhances recombination at two distinct sites in yeast rDNA. Mol. Microbiol.

[51] Ide S, Miyazaki T, Maki H, Kobayashi T (2010). Abundance of ribosomal RNA gene copies maintains genome integrity. Science.

[52] Kobayashi T, Ganley A R D (2005). Recombination Regulation by Transcription-Induced Cohesin Dissociation in rDNA Repeats. Science.

[53] Huang J, Brito I L, Villen J, Gygi S P, Amon A, Moazed D (2006). Inhibition of homologous recombination by a cohesin-associated clamp complex recruited to the rDNA recombination enhancer. Genes Dev.

[54] Burkhalter M D, Sogo J M (2004). rDNA enhancer affects replication initiation and mitotic recombination: Fob1 mediates nucleolytic processing independently of replication. Mol. Cell.

[55] Little R D, Platt T H, Schildkraut C L (1993). Initiation and termination of DNA replication in human rRNA genes. Mol. Cell. Biol.

[56] Gerber J K, Gogel E, Berger C, Wallisch M, Muller F, Grummt I, Grummt F (1997). Termination of mammalian rDNA replication: polar arrest of replication fork movement by transcription termination factor TTF-I. Cell.

[57] Lebofsky R, Bensimon A (2005). DNA Replication Origin Plasticity and Perturbed Fork Progression in Human Inverted Repeats. Mol. Cell. Biol.

[58] Ivessa A S, Lenzmeier B A, Bessler J B, Goudsouzian L K, Schnakenberg S L, Zakian V A (2003). The Saccharomyces cerevisiae helicase Rrm3p facilitates replication past nonhistone protein-DNA complexes. Mol. Cell.

[59] Nguyen V C, Clelland B W, Hockman D J, Kujat-Choy S L, Mewhort H E, Schultz M C (2010). Replication stress checkpoint signaling controls tRNA gene transcription. Nat. Struct. Mol. Biol.

[60] Prado F, Aguilera A (2005). Impairment of replication fork progression mediates RNA polII transcription-associated recombination. EMBO J.

[61] Azvolinsky A, Giresi P G, Lieb J D, Zakian V A (2009). Highly transcribed RNA polymerase II genes are impediments to replication fork progression in Saccharomyces cerevisiae. Mol. Cell.

[62] Tuduri S, Tourrière H, Pasero P (2009). Defining replication origin efficiency using DNA fiber assays. Chromosome Res.

[63] Koster D A, Crut A, Shuman S, Bjornsti M A, Dekker N H (2010). Cellular strategies for regulating DNA supercoiling: a single-molecule perspective. Cell.

[64] Wang J C (2002). Cellular roles of DNA topoisomerases: a molecular perspective. Nat. Rev. Mol. Cell Biol.

[65] Drolet M (2006). Growth inhibition mediated by excess negative supercoiling: the interplay between transcription elongation, R-loop formation and DNA topology. Mol. Microbiol.

[66] Gartenberg M R, Wang J C (1992). Positive supercoiling of DNA greatly diminishes mRNA synthesis in yeast. Proc. Natl. Acad. Sci. U S A.

[67] Travers A, Muskhelishvili G (2005). DNA supercoiling - a global transcriptional regulator for enterobacterial growth?. Nat. Rev. Microbiol.

[68] Fierro-Fernandez M, Hernandez P, Krimer D B, Stasiak A, Schvartzman J B (2007). Topological locking restrains replication fork reversal. Proc. Natl. Acad. Sci. U S A.

[69] Lambert S, Mizuno K, Blaisonneau J, Martineau S, Chanet R, Freon K, Murray J M, Carr A M, Baldacci G (2010). Homologous recombination restarts blocked replication forks at the expense of genome rearrangements by template exchange. Mol. Cell.

[70] Raghuraman M K, Winzeler E A, Collingwood D, Hunt S, Wodicka L, Conway A, Lockhart D J, Davis R W, Brewer B J, Fangman W L (2001). Replication Dynamics of the Yeast Genome. Science.

[71] Huvet M, Nicolay S, Touchon M, Audit B, d'Aubenton-Carafa Y, Arneodo A, Thermes C (2007). Human gene organization driven by the coordination of replication and transcription. Genome Res.

[72] Necsulea A, Guillet C, Cadoret J C, Prioleau M N, Duret L (2009). The Relationship between DNA Replication and Human Genome Organization. Mol. Biol. Evol.

[73] Cadoret J C, Meisch F, Hassan-Zadeh V, Luyten I, Guillet C, Duret L, Quesneville H, Prioleau M N (2008). Genome-wide studies highlight indirect links between human replication origins and gene regulation. Proc. Natl. Acad. Sci. U S A.

[74] Maric C, Prioleau M-N (2010). Interplay between DNA replication and gene expression: a harmonious coexistence. Curr. Opin. Cell Biol.

[75] Cayrou C, Coulombe P, Vigneron A, Stanojcic S, Ganier O, Peiffer I, Rivals E, Puy A, Laurent-Chabalier S, Desprat R, Mechali M (2011). Genome-scale analysis of metazoan replication origins reveals their organization in specific but flexible sites defined by conserved features. Genome Res.

[76] Nakayasu H, Berezney R (1989). Mapping replicational sites in the eucaryotic cell nucleus. J. Cell Biol.

[77] Jackson D A, Hassan A B, Errington R J, Cook P R (1993). Visualization of focal sites of transcription within human nuclei. EMBO J.

[78] Spector D L, Fu X D, Maniatis T (1991). Associations between distinct pre-mRNA splicing components and the cell nucleus. EMBO J.

[79] Wei X, Samarabandu J, Devdhar R S, Siegel A J, Acharya R, Berezney R (1998). Segregation of transcription and replication sites into higher order domains. Science.

[80] Cook P R (1999). The organization of replication and transcription. Science.

[81] Helleday T (2010). Homologous recombination in cancer development, treatment and development of drug resistance. Carcinogenesis.

[82] Beletskii A, Bhagwat A S (1996). Transcription-induced mutations: increase in C to T mutations in the nontranscribed strand during transcription in Escherichia coli. Proc. Natl. Acad. Sci. USA.

[83] Blackwell T K, Moore M W, Yancopoulos G D, Suh H, Lutzker S, Selsing E, Alt F W (1986). Recombination between immunoglobulin variable region gene segments is enhanced by transcription. Nature.

[84] Jung S, Rajewsky K, Radbruch A (1993). Shutdown of class switch recombination by deletion of a switch region control element. Science.

[85] Storb U, Peters A, Klotz E, Rogerson B, Hackett J (1996). The mechanism of somatic hypermutation studied with transgenic and transfected target genes. Semin. Immunol.

[86] Gottipati P, Cassel T N, Savolainen L, Helleday T (2008). Transcription-associated recombination is dependent on replication in Mammalian cells. Mol. Cell. Biol.

[87] Savolainen L, Helleday T (2009). Transcription-associated recombination is independent of XRCC2 and mechanistically separate from homology-directed DNA double-strand break repair. Nucleic Acids Res.

[88] Wellinger R E, Prado F, Aguilera A (2006). Replication fork progression is impaired by transcription in hyperrecombinant yeast cells lacking a functional THO complex. Mol. Cell. Biol.

[89] Jimeno S, Rondon A G, Luna R, Aguilera A (2002). The yeast THO complex and mRNA export factors link RNA metabolism with transcription and genome instability. EMBO J.

[90] Strasser K, Masuda S, Mason P, Pfannstiel J, Oppizzi M, Rodriguez-Navarro S, Rondon A G, Aguilera A, Struhl K, Reed R, Hurt E (2002). TREX is a conserved complex coupling transcription with messenger RNA export. Nature.

[91] Gómez-González B, García-Rubio M, Bermejo R, Gaillard H, Shirahige K, Marín A, Foiani M, Aguilera A (2011). Genome-wide function of THO/TREX in active genes prevents R-loop-dependent replication obstacles. EMBO J.

[92] Yu K, Chedin F, Hsieh C L, Wilson T E, Lieber M R (2003). R-loops at immunoglobulin class switch regions in the chromosomes of stimulated B cells. Nat. Immunol.

[93] Roy D, Yu K, Lieber MR (2008). Mechanism of R-loop formation at immunoglobulin class switch sequences. Mol. Cell Biol.

[94] Huertas P, Aguilera A (2003). Cotranscriptionally formed DNA:RNA hybrids mediate transcription elongation impairment and transcription-associated recombination. Mol. Cell.

[95] Huertas P, Garcia-Rubio M L, Wellinger R E, Luna R, Aguilera A (2006). An hpr1 point mutation that impairs transcription and mRNP biogenesis without increasing recombination. Mol. Cell. Biol.

[96] Li X, Manley J L (2005). Inactivation of the SR protein splicing factor ASF/SF2 results in genomic instability. Cell.

[97] Rossi F, Labourier E, Forne T, Divita G, Derancourt J, Riou J F, Antoine E, Cathala G, Brunel C, Tazi J (1996). Specific phosphorylation of SR proteins by mammalian DNA topoisomerase I. Nature.

[98] Paulsen R D, Soni D V, Wollman R, Hahn A T, Yee M C, Guan A, Hesley J A, Miller S C, Cromwell E F, Solow-Cordero D E, Meyer T, Cimprich K A (2009). A genome-wide siRNA screen reveals diverse cellular processes and pathways that mediate genome stability. Mol. Cell.

[99] Houlard M, Artus J, Leguillier T, Vandormael-Pournin S, Cohen-Tannoudji M (2011). DNA-RNA hybrids contribute to the replication dependent genomic instability induced by Omcg1 deficiency. Cell Cycle.

[100] Gomez-Gonzalez B, Felipe-Abrio I, Aguilera A (2009). The S-phase checkpoint is required to respond to R-loops accumulated in THO mutants. Mol. Cell. Biol.

[101] Artus J, Vandormael-Pournin S, Frodin M, Nacerddine K, Babinet C, Cohen-Tannoudji M (2005). Impaired mitotic progression and preimplantation lethality in mice lacking OMCG1, a new evolutionarily conserved nuclear protein. Mol. Cell. Biol.

[102] Shaw N N, Arya D P (2008). Recognition of the unique structure of DNA:RNA hybrids. Biochimie.

[103] Mischo H E, Gomez-Gonzalez B, Grzechnik P, Rondon A G, Wei W, Steinmetz L, Aguilera A, Proudfoot N J (2011). Yeast Sen1 helicase protects the genome from transcription-associated instability. Mol. Cell.

[104] Skourti-Stathaki K, Proudfoot N J, Gromak N (2011). Human Senataxin Resolves RNA/DNA Hybrids Formed at Transcriptional Pause Sites to Promote Xrn2-Dependent Termination. Mol. Cell.

[105] Aygün O, Svejstrup J. Q (2010). RECQL5 helicase: Connections to DNA recombination and RNA polymerase II transcription. DNA Repair.

[106] Li M, Xu X, Liu Y (2011). The Set2-RPB1 Interaction Domain of Human RECQ5 Is Important for Transcription-Associated Genome Stability. Mol. Cell. Biol.

[107] Kanagaraj R, Huehn D, MacKellar A, Menigatti M, Zheng L, Urban V, Shevelev I, Greenleaf A L, Janscak P (2010). RECQ5 helicase associates with the C-terminal repeat domain of RNA polymerase II during productive elongation phase of transcription. Nucleic Acids Res.

[108] Halazonetis T D, Gorgoulis V G, Bartek J (2008). An oncogene-induced DNA damage model for cancer development. Science.

[109] Tuduri S, Crabbe L, Tourriere H, Coquelle A, Pasero P (2010). Does interference between replication and transcription contribute to genomic instability in cancer cells?. Cell Cycle.

